# Identification and validation of immune‐related lncRNA prognostic signatures for melanoma

**DOI:** 10.1002/iid3.468

**Published:** 2021-06-02

**Authors:** Bo Xiao, Liyan Liu, Aoyu Li, Pingxiao Wang, Cheng Xiang, Hui Li, Tao Xiao

**Affiliations:** ^1^ Department of Orthopedics The Second Xiangya Hospital of Central South University Changsha Hunan China

**Keywords:** immune, lncRNAs, melanoma, prognosis, TCGA

## Abstract

**Introduction:**

Melanoma is a highly aggressive malignant skin tumor as well as the primary reason for skin cancer‐specific deaths. We first identified immune‐related long noncoding RNA (lncRNA) prognostic signature and found potential immunotherapeutic targets for melanoma cancer.

**Methods:**

RNA‐seq data and clinical features of melanoma samples were obtained from The Cancer Genome Atlas. Samples of melanoma were randomly assigned to the training and testing cohort. The immune‐related lncRNA signature was then obtained via using univariate, LASSO, and multivariate Cox analysis of patients in the training cohort. Eight significant immune‐related lncRNA signature was then subsequently obtained through correlation analysis between immune‐related genes and lncRNAs. The association between risk score and immune cell infiltration was finally assessed using TIMER and CIBERSORT.

**Results:**

Three hundred and fifty‐six immune‐related lncRNAs were obtained. Among them, eight immune‐related lncRNAs were identified to build a prognostic risk signature model. The model's performance was then confirmed using the Kaplan–Meier curves, risk plots, and time‐dependent receiver‐operating characteristic curves in the training cohort. The risk score was identified and confirmed as an independent prognostic factor through univariate and multivariate Cox regression analyses. These results were further verified in the testing and whole cohorts. CIBERSORT algorithm showed that the infiltration levels of T cells CD8, M1 macrophages, plasma cells, T cells CD4 memory activated, T cells gamma delta, and mast cells activated were significantly lower in the high‐risk group while the infiltration level of macrophages M0 was significantly lower in the low‐risk group.

**Conclusion:**

The immune‐related lncRNA signature offers prognostic markers and potential immunotherapeutic targets for melanoma.

## Graphical abstract

We applied univariate Cox, LASSO, and multivariate Cox regression analysis to identify immune‐related lncRNAs with remarkable prognostic potential and constructed a multiple immune‐related lncRNAs signature to predict melanoma' prognosis. lncRNA signatures of immune‐related offers prognostic markers and potential immunotherapeutic targets for melanoma.

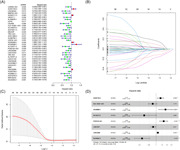

## 1 INTRODUCTION

Melanoma is an aggressive, melanocytic tumor characterized by poor prognosis in the metastatic stage. It is the most malignant skin cancer.^1^, ^2^ It accounts for nearly 1.7% (232,100) of newly diagnosed primary malignant cancers and about 0.7% (55,500) of deaths every year across the world.^3^ Currently, the incidence rates of melanoma are still increasing sharply.^4^ Cognizant to this, it is essential to identify novel prognostic markers for melanoma that could lead to better interventions for patients by providing novel therapeutic targets for personalized treatment regimes.

Long noncoding RNAs (lncRNAs) are RNAs with nonprotein coding that are usually over 200 nucleotides long. However, they have an indispensable role in tumor progression.^5^ They exhibit vital characteristics in tumor immunity, for example, immune cell migration, infiltration, antigen release, immune activation as well as antigen presentation.^6^, ^7^ For example, lncRNA SNHG1 is associated with the progression and differentiation of Treg cells in breast cancer and immune escape.^8^ Similarly, lncRNA TIM‐3 has been proved to be correlated with a decrease of antitumor immunity in hepatocellular carcinoma (HCC).^9^ Recent studies postulate that lncRNAs can act as novel prognostic biomarkers in melanoma.^10^, ^11^ Though Yang et al.^12^ reported that the value of lncRNA signatures in predicting melanoma prognosis. The studies on immune‐related lncRNA signatures in melanoma prognosis are still lacking.

Herein, a signature model comprising eight immune‐related lncRNAs for overall survival (OS) was first constructed utilizing uni‐ and multivariate, and LASSO Cox regression analysis to assess the value of immune‐related lncRNAs in melanoma prognosis. The samples were further assigned into two groups (low‐risk vs. high‐risk) based on the median risk score. Besides, we calculated the proportion of 22 immune cells in the training cohort using CIBERSORT algorithms to analyze the association between the differential proportion of immune cells and the risk score.

## 2 MATERIALS AND METHODS

### 2.1 Data collection

RNA sequencing fragments per kilobase per million (FPKM) for melanoma and the relevant clinical features were download from The Cancer Genome Atlas (TCGA) program. The merge script of perl and Ensembl database were used to combine RNA‐seq results into a matrix of gene symbols. The data set contained 471 melanoma tissues and one adjacent normal tissue. Samples without complete survival data as well as those whose OS was less than or equal to 30 days were removed from the study. Finally, 446 samples were included in the subsequent analysis.

### 2.2 Distinction of immune‐related lncRNAs

Immune‐related genes (IRGs) were downloaded from the Import Shared Data. Pearson's correlation analysis was then used to differentiate between expressed IRGs and lncRNA to identify immune‐related lncRNAs in melanoma samples (correlation coefficient >0.5, *p* < .001). We randomly divided the melanoma samples into the training and testing cohorts using the R package “caret.” The expression levels of immune‐related lncRNAs were further applied to build the prognostic model in the training cohort.

### 2.3 Establishment and verification of immune‐related lncRNAs signature associated with prognosis

Univariate Cox regression analysis was employed to opt immune‐related lncRNAs with significant correlation to overall survival of melanoma patients in training cohort. The LASSO regression analysis was then applied to determine the significance of the results obtained from univariate Cox analysis. Multivariate Cox regression analysis was subsequently applied to optimize the prognostic model of the results of LASSO analysis. The melanoma prognostic signature was finally constructed based on the immune‐related lncRNAs and their relevant coefficients result from multivariate Cox analysis. The formula used was: risk score = ∑*i*coefficient (lncRNA*i*) × expression (lncRNA*i*). The “survival,” “glmnet,” and “survminer” package in R were applied for univariate, LASSO and multivariate Cox regression analysis, respectively. The training and testing cohorts were further classified into two groups (high‐risk vs. low‐risk) based on the median risk score. Kaplan–Meier survival curves were drawn utilizing the “survival” package. Finally, time‐dependent receiver‐operating characteristic (ROC) analysis was used to explore the prognostic value of immune‐related lncRNA signature utilizing the “survivalROC” package.

### 2.4 Assessment of the immune‐related LncRNA signature as independent prognostic factor in melanoma patients

Both uni‐ and multivariate Cox regression analyses were employed to explore the clinical information in three cohorts. The information included the risk score, gender, age, and stage of disease.

### 2.5 Association analysis of immune cell infiltration

Immune infiltration data of B cells, CD4^+^ T cells, CD8^+^ T cells, dendritic cells, macrophages, and neutrophils were downloaded from the tumor immune estimation resource (TIMER) database, and the association between risk scores and immune infiltration was assessed by Pearson's correlation analysis.

### 2.6 CIBERSORT and differential content of immune cells in the two risk groups

CIBERSORT was applied to count the proportion of 22 immune cells in all samples of the training cohort given the importance of tumor immune cells infiltration in the tumor microenvironment. Those with significant differential contents of immune cells (*p* < .05) were used for further analysis. The differential content of immune cells in the two groups (low‐risk and high‐risk) was further compared utilizing the Wilcoxon rank‐sum test. A heatmap was then applied to show the differential proportion of immune cells in two risk groups. The color green indicated that the infiltrating levels were high while red indicated that the infiltrating levels were low.

## 3 RESULTS

### 3.1 Distinction of immune‐related lncRNAs

The 446 melanoma patients were randomly assigned into the training (224) and testing cohort (222) by using the R package “caret.” In both cohorts, 356 immune‐related lncRNAs were identified.

### 3.2 Establishment and validation of immune‐related lncRNAs signature

**Figure 1:**
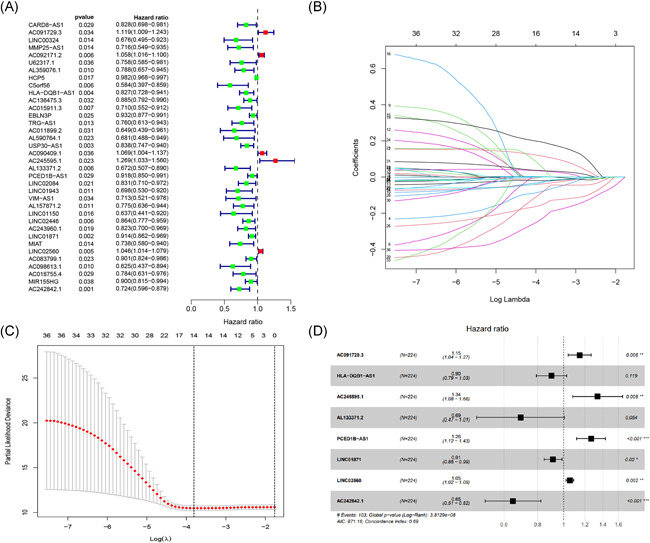
Distinction of immune‐related lncRNAs correlated with melanoma prognosis: univariate (A), LASSO (B, C), and multivariate Cox regression analysis (D). lncRNA, long noncoding RNA

The univariate Cox regression analysis was used to determine if there were any associations between the 356 immune‐related lncRNAs and OS of melanoma samples. From this analysis, 36 immune‐related lncRNAs were found to have a significant association with OS (Figure 1A). Further analysis of the 36 immune‐related lncRNAs through LASSO regression analysis led to the distinction of 14 immune‐related lncRNAs with the highest association with OS (Figure 1B,C). From the 14, 8 immune‐related lncRNAs were determined utilizing the multivariate Cox regression analysis and applied to build the prognostic model (Figure 1D). The eight immune‐related lncRNAs were: AC091729.3, HLA‐DQB1‐AS1, AC245595.1, AL133371.2, PCED1B‐AS1, LINC01871, LINC02560, and AC242842.1. The risk score was calculated according to the following formula; risk score = (0.13759866* AC091729.3 expression) + (−0.1033397* HLA‐DQB1‐AS1 expression) + (0.29162307* AC245595.1 expression) + (−0.3700152* AL133371.2 expression) + (0.23472527*PCED1B‐AS1 expression) + (−0.0904253* LINC01871 expression) +(0.05272196* LINC02560 expression) + (−0.4339273* AC242842.1 expression).

**Figure 2:**
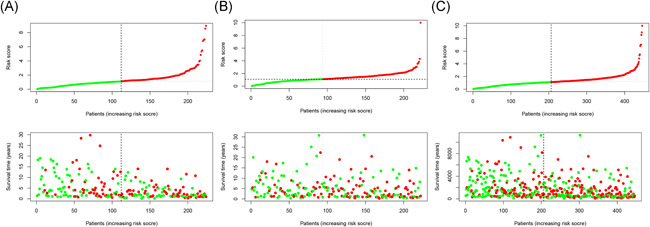
Risk score and survival status analysis (A–C) of melanoma in training, testing, and the whole cohort

**Figure 3:**
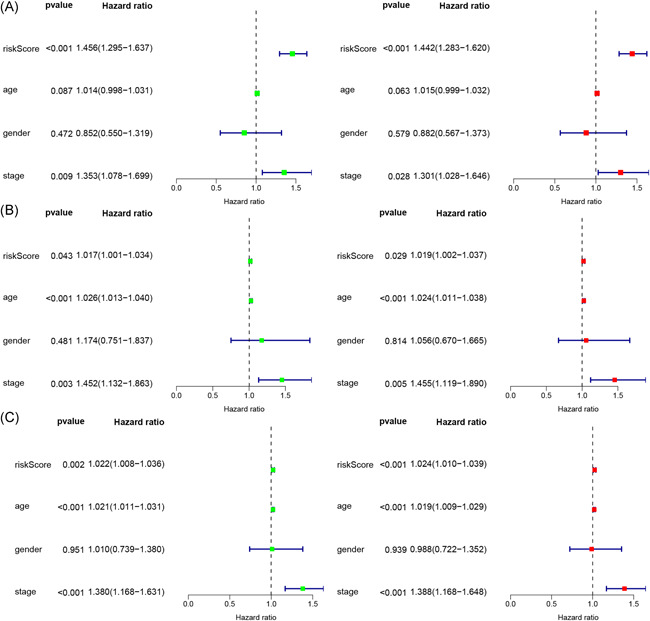
Uni‐ and multivariate Cox regression analysis of risk score, age, gender, and tumor stage in training (A), testing (B), and the whole (C) cohort

**Figure 4:**
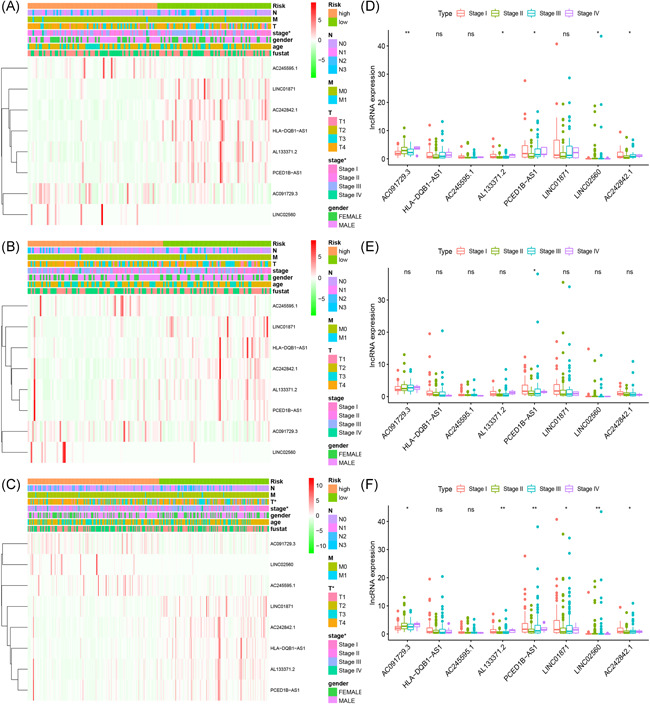
Association between risk score and clinical features of melanoma in training (A), testing (B), and whole (C) cohort. The expression levels of eight immune‐related lncRNAs in different stages from the training (D), testing (E), and whole (F) cohort. *p < .05, **p < .01, ***p < .001. lncRNA, long noncoding RNA

**Figure 5:**
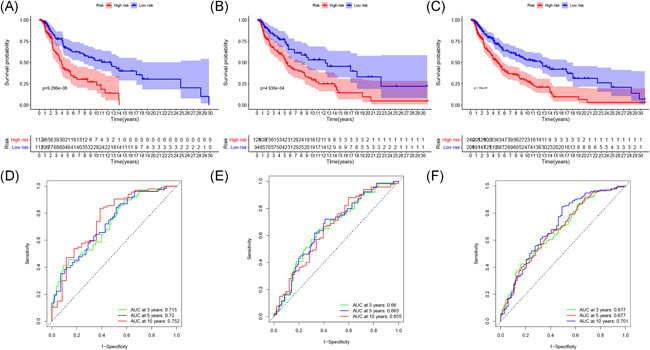
K–M survival analysis (A–C) and ROC curves (D–F) at 3, 5, and 10 years post diagnosis of immune‐related lncRNA signature in melanoma in training, testing, and whole cohort. lncRNA, long noncoding RNA; ROC, time‐dependent receiver‐operating characteristic

Patients in both cohorts were further divided into two groups (high‐risk vs. low‐risk) on the foundation of median risk score (Figure 2). Patients in high‐risk groups were found to have the worst survival. Figure 3A–C displays the heatmaps of the training, testing, and the whole cohort showing the expression levels of eight immune‐related lncRNAs in the two groups. Immune‐related lncRNAs that correlated with poor prognosis were: AC091729.3, AC245595.1, and LINC02560. The three lncRNAs were highly expressed in patients of the high‐risk group (Figure 4A–C). On the other hand, lncRNAs associated with a good prognosis were HLA‐DQB1‐AS1, AL133371.2, LINC01871, PCED1B‐AS1, and AC242842.1. The five lncRNAs were highly expressed in patients belonging to the low‐risk group (Figure 4A–C). The K–M curves further indicated that the low‐risk melanoma patients had a significantly longer survival in all cohorts (Figure 5A–C). Further to this, ROC curves were used to demonstrate the precision of the eight immune‐related lncRNA signatures in predicting the OS of melanoma patients at 3, 5, and 10 years postdiagnosis. The areas under the ROC (AUC) values were 0.715, 0.72, and 0.752 at 3, 5, and 10 years post diagnosis in the training cohort. And AUC values were over 0.66 at 3, 5, and 10 years post diagnosis in the testing and whole cohort (Figure 5D–F). This showed that the eight immune‐related lncRNAs had a favorable capacity in predicting the OS of melanoma.

### 3.3 Distinction of independent prognostic factors

Both univariate Cox and multivariate Cox regression analysis showed that the risk score and stage of disease were the independent prognostic factors for OS of melanoma in the training cohort (Figure 3A). The risk score, stage of disease, and age were the independent prognostic factors in the testing cohort (Figure 3B). Similarly, the risk score, age, and stage of the disease were also the independent prognostic factors in the whole cohort (Figure 3C). These results further illustrated that the risk score and stage of disease were the most significant independent prognostic factors for OS of melanoma.

### 3.4 Correlation between clinical features and immune‐related lncRNA signatures in melanoma patients

The melanoma samples in the training, testing, and whole cohort were first assigned into two groups (high‐risk vs. low‐risk) based on the median risk score (Figure 3A–C). The clinical information of melanoma patients included gender, age, stage of disease, tumor invasion (T), lymph node (N), and metastasis (M). We found the risk score was significantly associated with the stage of disease and T in the training and whole cohort. The results demonstrated that the eight immune‐related lncRNAs had a significant correlation with the stage of the disease. In addition, the expression of AC091729.3, AL133371.2, PCED1B‐AS1, LINC02560, and AC242842.1 was significantly different at different tumor stages in the training and whole cohort (Figure 4D,F). However, the expression of PCED1B‐AS1 was significantly different at different tumor stages in the testing cohort (Figure 4E). Moreover, the expression of AC091729.3 becomes higher in the training and whole cohort as the tumor stage continued to increase (Figure 4D,F).

### 3.5 Correlation between immune‐related lncRNA signatures and infiltration of immune cells

**Figure 6:**
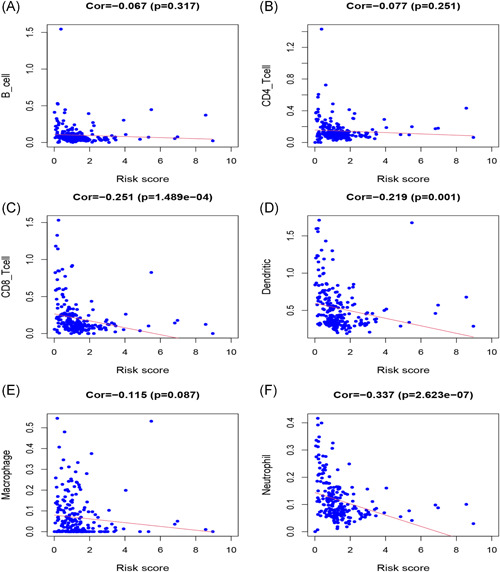
Association between eight immune‐related lncRNA for melanoma and infiltration of immune cells: B cells (A), CD4+ T cells (B), CD8+ T cells (C), dendritic cells (D), macrophages (E), and neutrophils (F). lncRNA, long noncoding RNA

The association between the eight lncRNA signatures associated with tumor immunity and six immune cells downloaded from the TIMER database was further analyzed to determine the correlation between eight immune‐related lncRNA signatures and infiltration of immune cells. The association values of dendritic cells, B cell, CD4^+^ T cells, CD8^+^ T cells, macrophages, and neutrophils with risk score were: −0.219, −0.067, −0.077, −0.251, −0.115, and −0.337, respectively (Figure 6A–F). These values indicated that the infiltration of the six immune cells was negatively associated with the prognosis of melanoma. This was particularly the case for CD8^+^T cells, neutrophils, and dendritic cells (*p* < .05). These results further demonstrated that the eight lncRNA signatures of melanoma were associated with infiltration of the six immune cells.

### 3.6 Differential content of tumor immune cells infiltration in two groups

**Figure 7:**
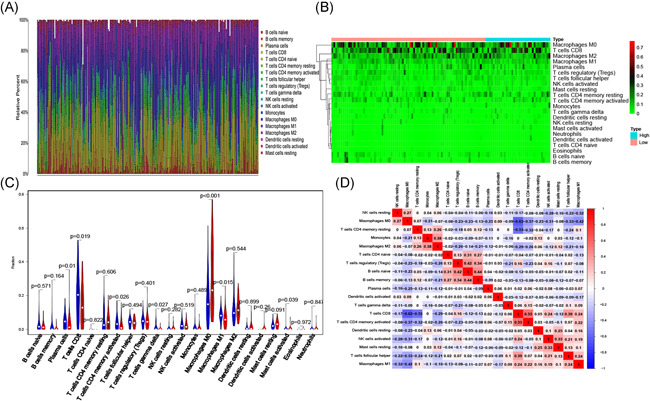
The proportion (A) and heatmap (B) of 22 immune cells in the training cohort. The comparison of 22 immune cells between two groups (low‐risk (green) vs. high‐risk (red)) (C). Correlation analysis of 22 immune cells (D)

The differential content of 22 immune cells in two groups was analyzed based on the association between eight immune‐related lncRNAs and six immune cells (Figure 7A). The heatmaps revealed the differential infiltration of immune cells in the two groups. Color green indicated that the infiltrating levels were high while red indicated that the infiltrating levels were low (Figure 7B). The Wilcoxon rank‐sum test further demonstrated that the density of the significantly low infiltrating immune cells was similar in the high‐risk group. The immune cells included the plasma cells (*p* = .01), T cells CD8 (*p* = .019), T cells CD4 memory activated (*p* = .026), T cells gamma delta (*p* = .027), mast cells activated (*p* = .039), and M1 macrophages (*p* = .015) (Figure 7C). Subsequently, correlation analysis was further used to illustrate the coexpression patterns of immune cells. As shown, T cells CD8 and macrophages M0 (*p* = −.63) and T cells CD4 memory resting (*p* = −.53) indicated a good negative correlation. Besides, several immune cells showed good positive correlation, such as B cells naive and T cell regulatory (*p* = .42) and B cells memory (*p* = .44) (Figure 7D).

## 4 DISCUSSION

Melanoma is a highly aggressive, melanocytic tumor characterized by a bad prognosis in the metastatic stage. It is the most malignant skin cancer.^1^, ^2^ The 5‐year survival rate of the majority of patients is about 90%. However, the survival rate of a third of melanoma patients with metastasis during diagnosis decrease by 5%–10%.^13^, ^14^ The mechanisms underlying melanoma development, progression, and metastasis remain unclear. It is critical to offer novel prognostic markers and provide novel therapeutic targets for melanoma.

Multiple studies have proved that cell immune responses play vital roles in the progression of cancer and thus are potential factors that influence cancer prognosis.^15^, ^16^ LncRNAs have traction for their roles in tumorigenesis, progression, and migration through competing endogenous RNA networks.^17^, ^18^ LncRNAs are involved in numerous biological processes, inflammation, autophagy, metabolism, and immune responses.^7^, ^19^, ^20^ Recent studies proved that lncRNAs act as vital regulators in the immune response of cancer. For example, lncRNA SNHG1 is involved in the progression of differentiation of Treg cells in breast cancer and immune escape.^8^ Similarly, lncRNA TIM‐3 has been proved to be correlated with a decrease of antitumor immunity in HCC.^9^ In the same line, lncRNA SNHG12 plays a vital role in evading immune‐mediated attacks and enhancing the immune response of tumor cells.^21^ As such, immune‐related lncRNAs are potential therapeutic targets and are invaluable in cancer prognosis. Despite these exciting reports, the role of immune‐related lncRNAs as effective biomarkers to predict survival in melanoma patients is still lacking.

Herein, 356 immune‐related lncRNAs were identified through correlation analysis of lncRNAs and IRGs. Eight immune‐related lncRNAs were correlated with OS in the training cohort through univariate, LASSO, and multivariate Cox regression analyses. The eight immune‐related lncRNAs were then applied to build the prognostic model to predict the OS of melanoma in the training cohort. The patients were further assigned into two groups on the foundation of the median risk score. Significant differences in OS among the two groups were only being found in the training cohort. Moreover, the predictive capability of the immune‐related signature was favorable at 3 years (AUC = 0.715), 5 years (AUC = 0.720), and 10 years (AUC = 0.752) post diagnosis in the training cohort, which is better than the results of previous study 2. The eight immune‐related lncRNA also had a similar predictive capability in the testing and whole cohort. This indicated that they had favorable practicability and repeatability for predicting OS.

Among the eight immune‐related lncRNAs, AC091729.3, AC245595.1, LINC02560, and PCED1B‐AS1 were risk‐associated, whereas AC242842.1, AL133371.2, HLA‐DQB1‐AS1, and LINC01871 were protective. Functions of some of the eight immune‐related lncRNAs have been probed and clarified. For example, PCED1B‐AS1 regulates proliferation and apoptosis of glioma by targeting the miR‐194‐5p/PCED1B axis.^22^ Moreover, it is a novel prognostic marker in glioblastoma that stimulates the Warburg effect, cell proliferation, and tumorigenesis via increasing the expression level of HIF‐1α.^23^ Similarly, LINC02560, HLA‐DQB1‐AS1, and LINC01871 are novel prognostic markers significantly associated with OS in squamous cell carcinoma of the tongue, lung adenocarcinoma, and breast cancer stem cell, respectively.^24^, ^25^, ^26^ Based on these facts, further studies on the underlying mechanisms of these lncRNAs in the prognosis of melanoma should be conducted.

Both uni‐ and multivariate Cox regression analysis revealed that the risk score and tumor stage were independent prognostic factors in melanoma patients. The number of samples in two groups (high‐risk vs. low‐risk) based on gender, age, tumor stage, T, N, and M in the three cohorts were further studied to determine the association between the immune‐related lncRNA signatures and clinical features of melanoma. Combined results revealed that there was a significant correlation between immune‐related lncRNA signatures and tumor stage. This correlation was highly significant in AC091729.3, AL133371.2, PCED1B‐AS1, LINC02560, and AC242842.1. This strongly indicated that the expression level of the five lncRNAs could be associated with the development of melanoma. As such, the underlying mechanism of action of the five lncRNAs in melanoma development should be further explored.

When the association of the infiltration of immune cells and risk score was conducted, plasma cells, T cells CD8, Mast cells activated, M1 macrophages, T cells CD4 memory activated, and T cells gamma delta were found to have significantly low densities in high‐risk groups. This result was similar to that of previous studies that also reported a decrease in infiltrating of CD8^+^ T cells, Mast cells, and CD4^+^ T cells in melanoma patients at high risk.^27^, ^28^, ^29^ Patients in the high‐risk group had a decrease in the infiltration of these immune cells which lead to a poor prognosis. Nonetheless, the underlying mechanisms of the eight immune‐related lncRNAs in the immune microenvironment of melanoma patients need to be further analyzed.

Inevitably, there some unavoidable limitations that need to be addressed. First, it was a retrospective study based on the public database. Second, the amount of data available from the public database is still limited, and there is no validation from other databases. So, multicenter studies and actual experiments are needed to verify our results before the immune‐related lncRNA signature can be applied in the clinic.

## 5 CONCLUSION

We first identified and confirmed an immune‐related lncRNA signature targeting IRGs in melanoma. The immune‐related lncRNA signature was validated to be an independent prognostic indicator for melanoma. Moreover, these eight lncRNAs maybe also provide novel diagnostic methods and potential immunotherapeutic targets for melanoma.

## CONFLICT OF INTERESTS

The authors declare that there are no conflict of interests.

## AUTHOR CONTRIBUTIONS

Bo Xiao designed the research study; Bo Xiao, Liyan Liu, and Aoyu Li performed the literature search and statistical analysis; and Bo Xiao, Aoyu Li, Pingxiao Wang, and Cheng Xiang interpreted the data and drafted the manuscript. Both Hui Li and Tao Xiao are the corresponding authors. Bo Xiao, Hui Li, and Tao Xiao critically revised the manuscript. All authors read and approved the final manuscript.
